# The effects of terlipressin and direct portacaval shunting on liver hemodynamics following 80% hepatectomy in the pig

**DOI:** 10.1042/CS20180858

**Published:** 2019-01-15

**Authors:** John S. Hammond, Fred Godtliebsen, Sonja Steigen, I. Neil Guha, Judy Wyatt, Arthur Revhaug, Dileep N. Lobo, Kim E. Mortensen

**Affiliations:** 1 Nottingham Digestive Diseases Centre and National Institute for Health Research (NIHR) Nottingham Biomedical Research Centre, Nottingham University Hospitals NHS Trust and University of Nottingham, Queen’s Medical Centre, Nottingham, U.K.; 2 Department of Hepato-Pancreatico-Biliary Surgery and Transplantation, Freeman Hospital, Newcastle upon Tyne, U.K.; 3 Department of Mathematics and Statistics, UiT – The Arctic University of Norway, Tromsø, Norway; 4 Institute of Medical Biology, UiT – The Arctic University of Norway, Tromsø, Norway; 5 Department of Clinical Pathology, The University Hospital of North Norway, Tromsø, Norway; 6 Department of Pathology, Leeds Teaching Hospitals NHS Trust, Leeds, U.K.; 7 Surgical Research Laboratory, Institute of Clinical Medicine, UiT – The Arctic University of Norway, Tromsø, Norway; 8 Department of Gastrointestinal Surgery, The University Hospital of North Norway, Tromsø, Norway; 9 MRC/ARUK Centre for Musculoskeletal Ageing Research, School of Life Sciences, University of Nottingham, Queen’s Medical Centre, Nottingham, U.K.

**Keywords:** post-resection liver failure, portacaval shunt, terlipressin

## Abstract

Liver failure is the major cause of death following liver resection. Post-resection portal venous pressure (PVP) predicts liver failure, is implicated in its pathogenesis, and when PVP is reduced, rates of liver dysfunction decrease. The aim of the present study was to characterize the hemodynamic, biochemical, and histological changes induced by 80% hepatectomy in non-cirrhotic pigs and determine if terlipressin or direct portacaval shunting can modulate these effects. Pigs were randomized (*n*=8/group) to undergo 80% hepatectomy alone (control); terlipressin (2 mg bolus + 0.5–1 mg/h) + 80% hepatectomy; or portacaval shunt (PCS) + 80% hepatectomy, and were maintained under terminal anesthesia for 8 h. The primary outcome was changed in PVP. Secondary outcomes included portal venous flow (PVF), hepatic arterial flow (HAF), and biochemical and histological markers of liver injury. Hepatectomy increased PVP (9.3 ± 0.4 mmHg pre-hepatectomy compared with 13.0 ± 0.8 mmHg post-hepatectomy, *P*<0.0001) and PVF/g liver (1.2 ± 0.2 compared with 6.0 ± 0.6 ml/min/g, *P*<0.0001) and decreased HAF (70.8 ± 5.0 compared with 41.8 ± 5.7 ml/min, *P*=0.002). Terlipressin and PCS reduced PVP (terlipressin = 10.4 ± 0.8 mmHg, *P*=0.046 and PCS = 8.3 ± 1.2 mmHg, *P*=0.025) and PVF (control = 869.0 ± 36.1 ml/min compared with terlipressin = 565.6 ± 25.7 ml/min, *P*<0.0001 and PCS = 488.4 ± 106.4 ml/min, *P*=0.002) compared with control. Treatment with terlipressin increased HAF (73.2 ± 11.3 ml/min) compared with control (40.3 ± 6.3 ml/min, *P*=0.026). The results of the present study suggest that terlipressin and PCS may have a role in the prevention and treatment of post-resection liver failure.

## Introduction

Post-resection liver failure (PLF) is a devastating complication that is resource intensive [1], carries considerable morbidity, and remains the primary cause of death following major liver resection [2]. Up to 90% of patients undergoing major (>50%) hepatectomy experience some degree of liver dysfunction [3]. This becomes clinically significant in half and progresses to PLF in up to 10% [2].

Risk factors identified for the development of PLF include extent of resection, presence of underlying parenchymal disease [2], elevated post-resection portal venous pressure (PVP) in non-cirrhotic patients [4], and pre-resection portal hypertension in cirrhotic patients [5]. Allard et al. [4] demonstrated that the risk of PLF and dying increased when post-resection PVP in non-cirrhotic patients increased above a threshold of 21–22 mmHg. The risk of PLF was negligible when PVP remained at normal levels (≤10 mmHg).

In porcine models of major liver resection, where post-resection PVP is modulated by portacaval shunting [6], mesocaval shunting [7,8], or by implantation of an adjustable vascular ring [9], the degree of post-resection liver dysfunction is reduced. Performing portacaval or mesocaval shunting in patients undergoing major liver resection adds complexity to the procedure; may increase morbidity through encephalopathy [10,11], could inhibit liver regeneration due to the diversion of hepatotropic factors [12] and requires an additional procedure to close the shunt once regeneration is complete. It is, therefore, desirable to explore strategies that reduce PVP without introducing additional morbidity peri/post-resection.

Tri-glycyl-lysine-vasopressin (terlipressin) is metabolized in the circulation to lysine-vasopressin, where its effects include reductions in PVP and portal venous flow (PVF) [13]. Terlipressin is widely used to treat complications of portal hypertension in patients with cirrhosis. It reduces rebleeding following acute variceal hemorrhage [14,15]; improves renal recovery in hepatorenal syndrome [16–20]; and reduces PVF following split graft liver transplantation [21]. Recent studies have also explored its effects after hepatectomy in rodents. Terlipressin reduced PVP following 90% hepatectomy [22], but had no effect on liver regeneration after 70% hepatectomy in rats [23]. In mice, terlipressin reduced PVP and increased liver regeneration after partial hepatectomy [24]. The effects of terlipressin on PVP after major hepatectomy in the absence of cirrhosis in a large animal model have not been reported.

The current study set out to characterize the hemodynamic, biochemical, and histological changes induced by 80% hepatectomy in non-cirrhotic pigs in a terminal anesthetic model and to determine if terlipressin or direct portacaval shunting (PCS) could reverse these effects. We hypothesized that terlipressin and PCS would reduce PVP and PVF and increase hepatic artery flow (HAF) post-hepatectomy.

## Methods

### Study design

The study was undertaken in three parts: an acute pilot study, an acute non-survival series, and a survival pilot study (Supplementary Figure S1). The acute pilot study provided preliminary data on hemodynamic and biochemical changes pre/post-hepatectomy±terlipressin or PCS, and determined the optimal terlipressin-dosing regimen under terminal anesthesia.

The acute series compared the hemodynamic effects of terlipressin or PCS pre/post-hepatectomy in pigs maintained under terminal anesthesia for up to 8 h post-hepatectomy. Pigs were randomized (sealed envelope drawn 1 week prior to surgery), to undergo hepatectomy alone (control); terlipressin followed by hepatectomy or PCS followed by hepatectomy. There was no sham group in this series. PVP, PVF, HAF, and mean arterial pressure (MAP) were recorded continuously. Arterial and portal venous blood and liver biopsies were taken at intervals throughout the series. Biopsies were also collected post-mortem.

In the survival pilot, pigs underwent 80% hepatectomy alone and were maintained for up to 7 days post-hepatectomy. There was no comparison group in the survival pilot. PVP and PVF were recorded and central/portal venous blood samples were taken daily. Biopsies were collected post-mortem. Data from the survival pilot are presented in the Supplementary Material.

### Animals

All protocols were approved by the Norwegian Animal Research Authority, conducted in compliance with and presented in accordance with the National Institute of Health’s *Guide for the Care and Use of Laboratory Animals* [25]. Based on the pilot series, we estimated that to demonstrate a 10% reduction in PVP (i.e. to reduce post-resection PVP to <10 mmHg) with terlipressin or PCS post-hepatectomy, eight animals were required per group. In total 40 castrate male Norwegian pigs (*Sus scrofa domesticus*, weight = 32.0 ± 5.9 kg) were used: 8 in the acute pilot, 24 in the acute series, and 8 in the survival pilot.

Anesthesia was administered using an established protocol developed previously within the group [26]. Pigs were pre-medicated with intramuscular ketamine (20 mg/kg) and atropine (1 mg). Anesthesia was induced with intravenous fentanyl (0.01 mg/kg) and isofluorane in oxygen (FiO_2_ = 0.5, Servo 900, Elema-Schönander/Siemens, Erlangan Germany) and maintained with intravenous fentanyl (0.02 mg/kg/h), midazolam (0.3 mg/kg/h), and isflourane in oxygen. Ceftriaxone (2 g) was given post-induction.

5-F catheters (CVK, Secalon T, Argon Critical Care Ltd., Singapore, Singapore) were placed in both internal jugular veins and left femoral artery (blood sampling and MAP). Intravenous fluids were delivered at 100 ml/h with boluses to maintain MAP > 50 mmHg, central venous pressure (CVP) 5–8 mmHg, and urine output > 0.5 ml/kg/h. If refractory hypotension developed (MAP <50 mmHg for >10 min despite volume replacement) norepinephrine (0.025 μg/kg/h) was commenced.

### Liver hemodynamic monitoring

Laparotomy was performed through a right-sided, reverse-L incision; 3-mm flow probes were placed around the left and right hepatic arteries and a 12-mm flow probe around the portal vein (Medistim, Oslo, Norway). A 6-F double lumen catheter was placed directly into the portal vein (Arrow International, Reading, U.S.A.) and secured with 5/0 polypropylene sutures. Calibrated transducers (Transpac 3, Abbott Critical Care Systems, Chicago, U.S.A.) were connected to an amplifier (Gould, 2800S, Ohio, U.S.A.). Pulsatile signals were displayed, digitized, and stored electronically.

### Terlipressin

In the terlipressin group (Glypressin®, donated by Ferring Pharmaceuticals, West Drayton, U.K.), a 2-mg intravenous terlipressin bolus was given 20 min pre-hepatectomy and an intravenous terlipressin infusion (0.5–1 mg/h) was commenced post-hepatectomy and continued for the duration of the experiment. No placebo was given in the control or PCS groups.

### Direct portacaval shunt

In the shunt group, a side-to-side direct PCS was sutured using continuous 5/0 polypropylene on the infrahepatic portion of the inferior vena cava, as described previously [6,27] with an increase in shunt diameter from 5 to 8 mm. Partial (side) clamping of the portal vein and inferior vena cava was required during PCS formation. Shunt patency was confirmed by demonstrating PVF reduction following clamp release and by direct inspection and measurement post-mortem. Hepatectomy was commenced 20 min after completion of PCS.

### Eighty percent hepatectomy

Eighty percent hepatectomy was undertaken as previously described [28,29] with minor modifications. The left hepatic artery, portal vein, and bile duct were ligated at the hilum. Segments II, III, IV, V, and VIII were resected *en bloc* with manual control of the vascular pedicle. The pedicle stump was oversewn with 2/0 polyglactin. Segment VI was resected by manual control of the vascular VI/VII pedicle and its venous branches oversewn with 2/0 polyglactin, to leave segments I and VII. Resected wet liver weights were recorded. An estimated remnant liver weight was calculated using the equation: remnant liver weight (g) = 0.025 × total body weight (g) − resected liver weight (g).

### Survival pilot study

In the survival pilot, tunneled single lumen 6-F Broviac catheters (Bard Access Systems Inc, Salt Lake City, U.S.A.) were placed in each internal jugular vein. The portal catheter and flow probe were tunneled laterally through the abdominal wall. A feeding gastrostomy (Cook Medical Inc., Bloomington, U.S.A.) was inserted. Lines/cables were secured with a protective vest (Lomir Biomedical Inc., Malone, U.S.A.). Fluids, analgesia, and antibiotics were given daily. Blood was taken and CVP, PVP, and PVF were recorded daily under sedation (midazolam 0.15 mg/kg) in the left-lateral position. HAF/MAP were not recorded and no pigs received/underwent terlipressin/PCS in the survival series.

### Post-mortem

At the end of each experiment, blood, liver, spleen, small bowel, and left kidney biopsies were collected. Probe/catheter positions were confirmed and the liver±shunt were weighed/measured.

### Biochemistry

Serum aspartate aminotransferase (AST), bilirubin, sodium, potassium, urea, creatinine, and plasma ammonia were measured using a cobas® c analyzer (Roche Diagnostics, Indianapolis, U.S.A.); international normalized ratio (INR) with an STA® prothrombin time assay kit (Diagnostica Stago SAS, Asnières sur Seine Cedex, France); serum lactate with an ABL 800 flex blood gas analyser (Radiometer Medical ApS, Brønshøj, Denmark); and Lysine-vasopressin with a (Lys^8^) vasopressin ELISA kit (Sigma–Aldrich, St. Louis, U.S.A.).

### Histological analysis

Liver, small bowel, splenic, and renal biopsies were divided and flash-frozen in liquid nitrogen and stored at −80°C or processed for histology, by fixing under vacuum in 10% neutral-buffered formalin for 24 h at 37°C and stored for up to 1 month. Histology samples were paraffin-embedded on a Shandon™ Excelsior™ ES tissue processor (Thermo Fisher Scientific Inc., Waltham, U.S.A.). Three millimeter sections were stained with Hematoxylin and Eosin (H & E).

### Hemodynamic analysis

Flow was compared using raw data (ml/min) and flow by liver weight (ml/min/g). The latter was calculated using the equation: flow/g (ml/min/g) = total flow (ml/min)/0.005 × body weight (g). HAF was the sum of left and right HAFs. Data are expressed as the mean ± S.D. unless otherwise stated. PVP, PVF, HAF, and MAP were analyzed with repeated-measures ANOVA, using data extracted from the real-time data material sampled over 10-min intervals and analyzed using IBM SPSS 22.0 for Mac OSX SPSS (IBM Corp., Armonk, NY). Differences were considered statistically significant at *P*<0.05.

## Results

### Pilot data and the effects of terlipressin and direct PCS pre-hepatectomy

Terlipressin and PCS reduced PVP and PVF if given/performed pre- or post-hepatectomy. To standardize the approach between groups, terlipressin-dosing and PCS were undertaken pre-hepatectomy. The pigs were maintained for up to 8 h post-hepatectomy, because in the acute pilot experiments there was typically a progressive deterioration in physiological parameters beyond 8 h.

**Figure 1:**
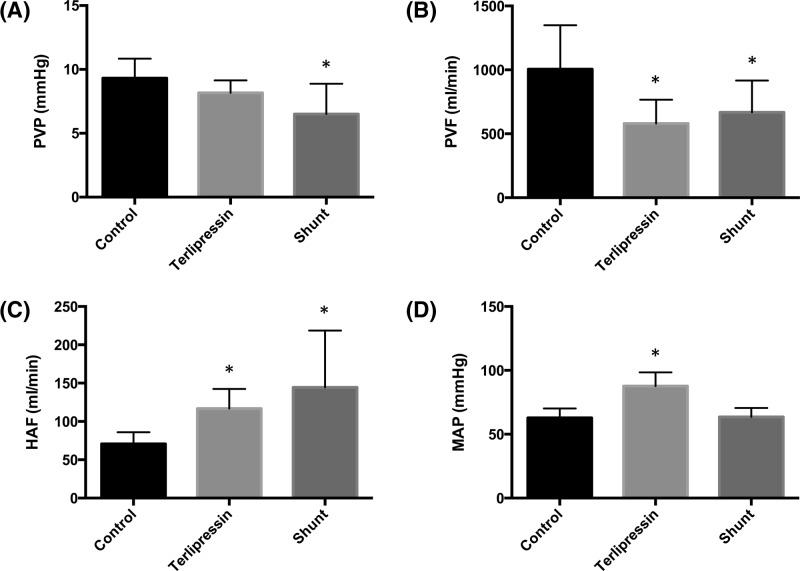
The effects of terlipressin and direct PCS on PVP, PVF, HAF, and MAP in normal liver (prior to 80% hepatectomy) Values represent mean ± S.D., *n*=8 per group. *P<0.05 compared with control (pre-terlipressin or pre-PCS) PVP, PVF, HAF, or MAP. (**A**) The effects of terlipressin or PCS on PVP in normal liver. Control compared with terlipressin, *P*=0.11. Control compared with PCS, *P*=0.009. (**B**) The effects of terlipressin or PCS on PVF in normal liver. Control compared with terlipressin, *P*=0.003. Control compared with PCS, *P*=0.017. (**C**) The effects of terlipressin or PCS on HAF in normal liver. Control compared with terlipressin, *P*=0.001. Control compared with PCS, *P*=0.012. (**D**) The effects of terlipressin or PCS on MAP prior to hepatectomy. Control compared with terlipressin, *P*<0.0001. Control compared with PCS, *P*=0.80. Abbreviation: MAP, mean arterial pressure.

Following 2 mg terlipressin pre-hepatectomy, PVP remained stable (Figure 1A), PVF decreased (Figure 1B), and HAF increased (Figure 1C). Following PCS, PVP, and PVF decreased and HAF increased. PCS patency was confirmed by a reduction in PVF from 988 ± 296 to 715 ± 252 ml/min. The reduction was similar between animals. There were no differences in PVP, PVF, or HAF between the terlipressin and PCS groups. MAP increased after terlipressin. PCS had no effect on MAP pre-hepatectomy (Figure 1D).

The segments II, III, IV, V, and VIII resection resulted in a 78.9 ± 2.3% hepatectomy, with an additional cuff of devascularized parenchyma at the base of segments II/VIII. The average time for hepatectomy was 37 ± 8 min. The average shunt diameter measured at post-mortem was 8 ± 1 mm.

### The effects of hepatectomy on liver and systemic hemodynamics and biochemistry

**Figure 2:**
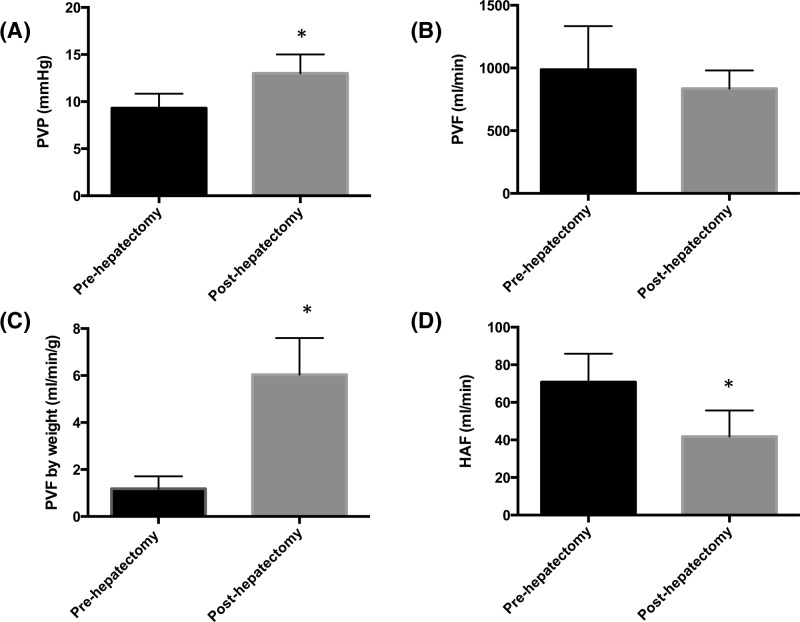
The effects of 80% hepatectomy on PVP, PVF (by liver weight), and HAF were assessed 30 min post-hepatectomy Values represent mean ± S.D., *n*=8 per group. *P<0.05 compared with pre-hepatectomy PVP, PVF, PVF by liver weight, or HAF. (**A**) The effects of 80% hepatectomy on PVP. Pre compared with post, *P*<0.0001. (**B**) The effects of 80% hepatectomy on PVF. Pre compared with post, *P*=0.22. (**C**) The effects of 80% hepatectomy on PVF by liver weight. Pre compared with post, *P*<0.0001. (**D**) The effects of 80% hepatectomy on HAF. Pre compared with post, *P*=0.002.

In the control group, PVP increased post-hepatectomy and remained elevated throughout the experiment (Figure 2A). There was no change in PVF post-hepatectomy (Figure 2B) although the PVF/g increased (Figure 2C). HAF decreased post-hepatectomy and remained lower throughout the experiment (Figure 2D).

In the PCS group, four pigs developed refractory hypotension within 3 h of hepatectomy necessitating norepinephrine. One pig in the control group required norepinephrine after 3 h. Norepinephrine was not required in the terlipressin group. CVP was maintained between 5 and 8 mmHg and urine output > 0.5 ml/kg/h throughout the experiment. Urine output increased in the terlipressin group compared with control and PCS groups.

**Figure 3:**
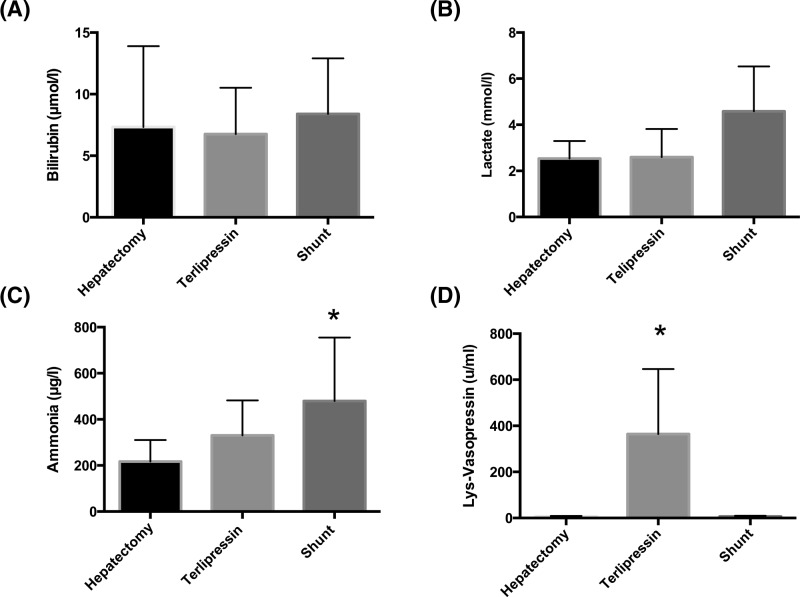
Serum bilirubin, serum lactate, plasma ammonia, and serum lys-vasopressin at 3 h post hepatectomy Values represent mean ± S.D., *n*=8 per group. *P<0.05 compared with control (hepatectomy alone) bilirubin, lactate, ammonia, or terlipressin. (**A**) Bilirubin at 3 h post-hepatectomy. Control compared with terlipressin, *P*=0.18. Control compared with PCS, *P*=0.31. (**B**) Lactate at 3 h post-hepatectomy. Control compared with terlipressin, *P*=0.37. Control compared with PCS, *P*=0.09. (**C**) Ammonia at 3 h post-hepatectomy. Control compared with terlipressin, *P*=0.11. Control compared with PCS, *P*=0.03. (**D**) Lys-vasopressin at 3 h post-hepatectomy. Control compared with terlipressin, *P*<0.0001. Control compared with PCS, *P*=0.50.

Figure 3A–D summarizes the biochemistry from the acute series. Sodium, potassium, urea, and creatinine (not shown) remained within normal limits in all groups throughout the series. Bilirubin (Figure 3A), lactate (Figure 3B), INR, and AST increased in all groups but no differences were detected between groups. Ammonia increased in all groups and was greater in the PCS group at 3 h compared with control (Figure 3C). Lys-vasopressin was detected in all groups. Levels remained at baseline in the control and PCS groups and increased in the terlipressin group (Figure 3D).

In the survival pilot 80% hepatectomy was undertaken with 100% at 1-day and 62% at 3-day survival. The pigs experienced significant morbidity (pain and ascites) post-hepatectomy. PVP increased and remained elevated up to day 5 post-hepatectomy (Supplementary Figure S2A). PVF initially decreased post-hepatectomy, then by 12 h PVF had increased from baseline and remained elevated up to day 5 (Supplementary Figure S2B). HAF was not measured in the survival experiments. Serum sodium, potassium, and urea were within normal limits throughout the survival pilot. Serum creatinine increased on day 1, and returned to baseline by day 2. Serum bilirubin (Supplementary Figure S2C) peaked on day 2. INR (Supplementary Figure S2D), AST (Supplementary Figure S2E), and ammonia (Supplementary Figure S2F) peaked on day 1. INR normalized by day 4. Bilirubin, AST, and ammonia remained elevated throughout the survival pilot.

### The effects of terlipressin and PCS on PVP post-hepatectomy

**Figure 4:**
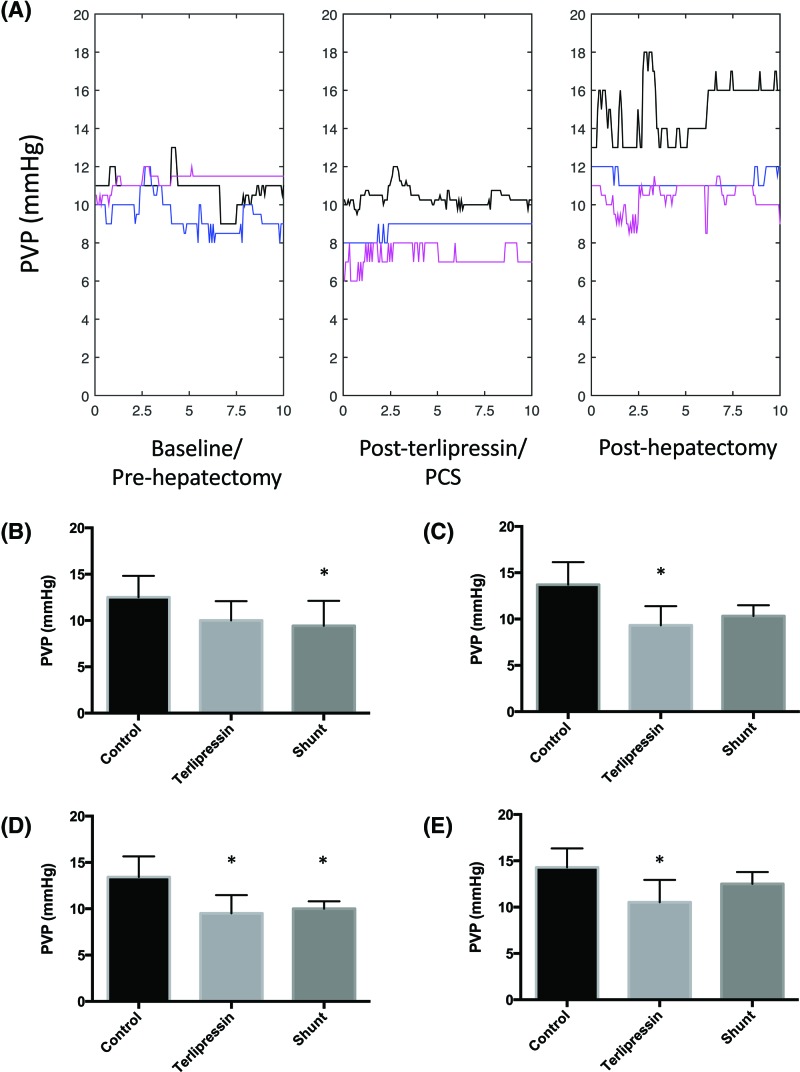
The effects of terlipressin and PCS on PVP following 80% hepatectomy (**A**) Values represent continuous median PVP for all pigs in each group sampled over representative 10-min intervals and processed using scale space analysis: pre-hepatectomy (left), post-terlipressin, or PCS but before hepatectomy (middle) and 1 h post-hepatectomy (right). Hepatectomy alone (black), terlipressin + hepatectomy (blue) and PCS + hepatectomy (pink). The x-axis represents time in minutes, Values in (**B**–**E**) represent mean ± S.D. of representative 10-min intervals of PVP sampled out to 6 h post hepatectomy, *n*=8 per group. *P<0.05 compared with control: (B) immediately post-hepatectomy (T_0_); (C) 1–2 h; (D) 3–4 h; (E) 5–6 h (Supplementary Table S1). Values represent median PVP of three 10-min intervals sampled for all pigs in each group processed using scale space analysis.

Figure 4A traces the continuous median PVP for representative measurements for all pigs in each group over 10-min intervals: baseline; pre-hepatectomy but post-terlipressin/PCS; and post-hepatectomy. There were no differences in baseline PVP between groups. PVP increased post-hepatectomy from baseline and remained elevated for the duration of the study. There was no difference in baseline and post-hepatectomy PVP in the terlipressin and PCS groups for the duration of the study.

Figure 4B–E summarizes the mean PVP of representative 10-min intervals sampled immediately post-hepatectomy and hourly throughout the acute series. Terlipressin reduced post-hepatectomy PVP within 1 h and its effects were sustained throughout the series when compared with the control group and not within the terlipressin group. PCS reduced post-hepatectomy PVP for up to 4 h post-hepatectomy when compared with the control group and not within the PCS group. There was no difference in PVP between the terlipressin and PCS groups throughout the series.

### The effects of terlipressin and PCS on PVF post-hepatectomy

**Figure 5:**
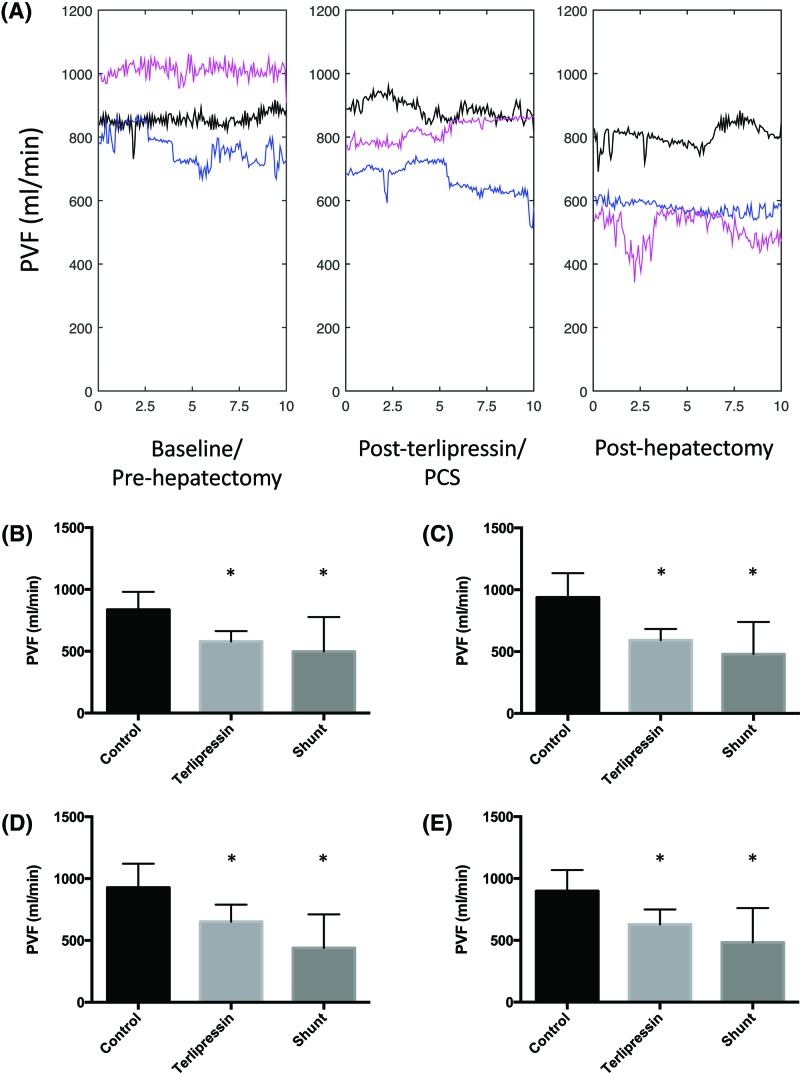
The effects of terlipressin and PCS on PVF following 80% hepatectomy (**A**) Values represent continuous median PVF for all pigs in each group sampled over representative 10-min intervals and processed using scale space analysis: pre-hepatectomy (left), post-terlipressin, or PCS but before hepatectomy (middle) and 1 h post-hepatectomy (right). Hepatectomy alone (black), terlipressin + hepatectomy (blue) and PCS + hepatectomy (pink). The x-axis represents time in minutes. Values in (**B**–**E**) represent mean ± S.D. of representative 10-min intervals of PVF sampled out to 6 h post-hepatectomy, *n*=8 per group. *P<0.05 compared with control: (B) immediately post-hepatectomy (T_0_); (C) 1–2 h; (D) 3–4 h; (E) 5–6 h (Supplementary Table S2).

Figure 5A traces the continuous median PVF for representative measurements for all pigs in each group over 10-min intervals. There were no differences in pre/post-hepatectomy PVF in the control, terlipressin (*P*=0.84), or PCS (*P*=0.21) groups. PVF/g increased in all groups post-hepatectomy (not shown).

Figure 5B–E summarizes the mean PVF for representative intervals sampled over the post-hepatectomy period. Terlipressin and PCS led to reductions in PVF throughout the series post-hepatectomy when compared with the control group and not within the terlipressin nor the PCS groups. There were no differences in PVF between the terlipressin and PCS groups throughout the series.

### The effects of terlipressin and PCS on HAF post-hepatectomy

**Figure 6:**
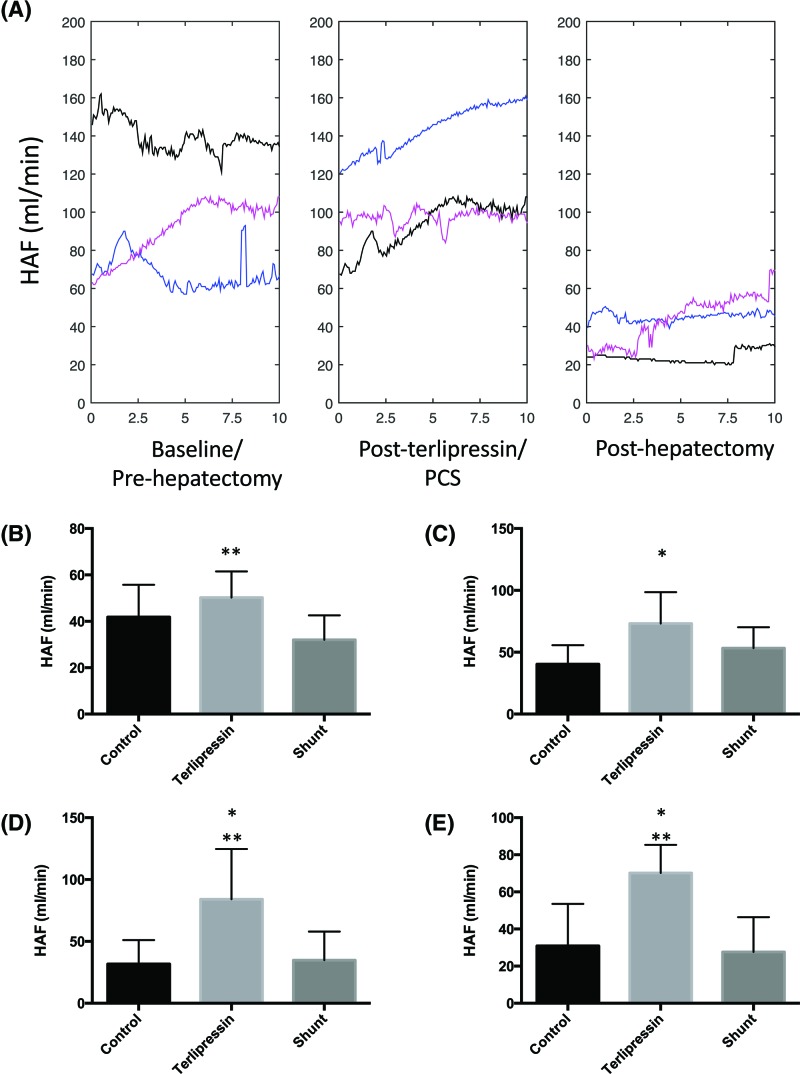
The effects of terlipressin and PCS on HAF following 80% hepatectomy (**A**) Values represent continuous median PVP for all pigs in each group sampled over representative 10-min intervals and processed using scale space analysis: pre-hepatectomy (left), post-terlipressin, or PCS but before hepatectomy (middle) and 1 h post-hepatectomy (right). Hepatectomy alone (black), terlipressin+hepatectomy (blue) and PCS+hepatectomy (pink). The x-axis represents time in minutes. Values in (**B**–**E**) represent mean ± S.D. of representative 10-min intervals of HAF sampled out to 6 h post-hepatectomy, *n*=8 per group. *P<0.05 compared with control and **P<0.05 compared with PCS: (B) immediately post-hepatectomy (T_0_); (C) 1–2 h; (D) 3–4 h; (E) 5-6 h (Supplementary Table S3).

Figure 6A traces the continuous median HAF for all pigs in each group over 10-min intervals. HAF decreased in the hepatectomy alone, terlipressin (*P*=0.003) and PCS (*P*=0.024) groups post-hepatectomy.

Figure 6B–E summarizes the mean HAF for representative intervals sampled over the post-hepatectomy period. Immediately post-hepatectomy HAF in the terlipressin group exceeded HAF in the PCS group but not the control. After 1 h HAF in the terlipressin group was greater than control and remained higher for up to 7 h post-hepatectomy. HAF in the terlipressin group also exceeded that of the PCS group for prolonged intervals post-hepatectomy. There was no difference in HAF between the PCS and control groups throughout the series.

### Histology

There were variations in the baseline liver tissue within and between groups, in terms of steatosis, hepatocyte staining, sinusoidal diameter, and presence of intra-sinusoidal mononuclear cells. All groups demonstrated extravasation of red cells 2 h post-hepatectomy with progressive portal edema (Supplementary Figure S3A) and neutrophil migration appearing 6 h post-hepatectomy. The extent of red cell extravasation and portal edema in the pigs receiving terlipressin was reduced at later time points compared with the control pigs. No proliferative markers were assessed in this acute study. It was not possible to quantitate these differences using image analysis. No evidence of splenic, kidney, or small bowel pathology was detected across the acute series.

In the survival pilot, up to 3 days post-hepatectomy post-mortem liver histology demonstrated variable venous congestion, sinusoidal dilatation, and sinusoidal mononuclear cell infiltration. No biliary changes were demonstrated up to day 3. There was evidence of hepatocyte and non-parenchymal cell regeneration. From days 4–7 sinusoidal dilatation and venous congestion persisted. In addition, there was evidence of inflammation, biliary injury [desquamation and infarction (Supplementary Figure S3B)] and steatosis (Supplementary Figure S3C). Regenerative changes were less evident than in earlier post-mortem specimens. All splenic biopsies from the survival pilot demonstrated venous congestion. Small bowel biopsies demonstrated bowel wall thickening. There was no evidence of kidney pathology.

## Discussion

The present study demonstrates that 80% hepatectomy in the pig increases PVP and PVF/g and reduces HAF, and that terlipressin and PCS attenuate these effects in a terminal anesthetic model. Although previous studies have demonstrated the effects of PCS on liver hemodynamics post-hepatectomy in pigs [6,7] and of terlipressin post-hepatectomy in rodents [22–24], this is the first study to report the effects of terlipressin on liver hemodynamics post-hepatectomy in a non-cirrhotic porcine model.

The effects of terlipressin and PCS were characterized using a terminal anesthetic model, previously developed within our group [30–32]. This enabled multiple, continuous pressure and flow measurements to be recorded simultaneously while minimizing morbidity in the study group. While it is feasible to measure liver and systemic hemodynamics at intervals post-hepatectomy in a survival setting [7,29], in our experience there is greater variability in PVP and PVF between pigs (due to the physiological instability that accompanies the ensuing PLF) and the animals are exposed to significant morbidity. The terminal anesthetic model allowed us to demonstrate continuous real-time physiology in the early phase post-hepatectomy. The limitation of this model is that it does not enable characterization of liver hemodynamics beyond 8 h.

In the survival pilot, after 80% hepatectomy PVP and PVF changes were sustained for 5 days and accompanied by significant liver dysfunction. This clinical course was comparable with existing studies [29]. Histology 1-week post-hepatectomy demonstrated hepatic sinusoidal dilatation, venous congestion, steatosis, and inflammation within a regenerating liver. There are limited reports of histological changes in pig liver after extended hepatectomy. Similar patterns may be observed in patients who develop small-for-size syndrome after split-liver transplantation, where liver biopsies taken within the first 10 days post-transplantation demonstrate venous congestion, sinusoidal injury, steatosis, and cholestasis [33]. With the exception of cholestasis (often a later change), these features were present in the survival pilot.

Previous studies have demonstrated that 80% hepatectomy in the pig increases PVP, leads to liver dysfunction and increases mortality [29]. In this terminal anesthetic study, 80% hepatectomy led to a PVP increase. Both terlipressin and PCS maintained PVP at pre-resection levels after 80% hepatectomy for the duration of the study. In addition, post-hepatectomy PVP was significantly lower than in the control group for up to 4 h in the PCS group and up to 6 h in the terlipressin group. If this effect was sustained in a survival model, terlipressin and PCS could have an impact on rates of liver dysfunction.

The post-hepatectomy PVP increase in pigs is less than the PVP increase following equivalent resections in patients [4], although their clinical course is comparable [29]. The difference in post-hepatectomy PVP between pig and human liver may be explained by variations in parenchymal compliance, venous outflow, and the presence of unreported parenchymal disease in patients undergoing major hepatectomy. The cause for variation in baseline tissue is unknown, but was not thought to have impacted on differences in liver hemodynamics between groups, as the pigs were randomized pre-operatively and there was no fibrosis or cirrhosis detected in the baseline liver biopsies.

The hepatic artery buffer response autoregulates liver blood flow. When PVF increases, HAF decreases and *vice versa* [34,35]. This was demonstrated pre-hepatectomy in normal liver where both terlipressin and PCS reduced PVF resulting in an increase in HAF [7].

Post-hepatectomy terlipressin reduced PVF and increased HAF compared with the control group. Vasopressin exerts a biphasic response on HAF. If infused directly into the hepatic artery vasopressin leads to hepatic artery vasoconstriction. When it is given systemically, vasopressin causes splanchnic vasoconstriction; reducing PVF, which in turn increases HAF, through the buffer response [36]. PCS reduced PVF and increased HAF pre-hepatectomy. Post-hepatectomy showed no difference in HAF as demonstrated when compared with control. Interpretation of the hemodynamic effects of PCS on HAF is difficult because 50% of pigs in the PCS group required norepinephrine, which is likely to have had a direct vasoconstrictive effects on the hepatic artery [37].

The decreased oxygen delivery that results from HAF reduction, together with the venous congestion that arises from increasing PVF/g, may induce hypoxia in the remnant liver, precipitating a cycle of inflammation and impaired regeneration, which could exacerbate liver dysfunction. This process may have parallels with ischemia–eperfusion injury [38].

Eighty percent hepatectomy+PCS caused hemodynamic instability that required supplementary fluids and norepinephrine 3–4 h post-hepatectomy. No pigs receiving terlipressin required norepinephrine. MAP increased significantly following administration of terlipressin. It is likely that terlipressin-induced arteriolar vasoconstriction augmented MAP, however the absence of information regarding cardiac index or vascular resistance, limits the ability to distinguish true terlipressin-induced changes.

Direct PCS was used as this had previously been demonstrated to modulate liver dysfunction in pigs after major hepatectomy [6]. The increased hemodynamic instability in the PCS group was an unexpected finding. While the duration of partial portal clamping was minimized during shunt formation, portal clamping is very poorly tolerated in pigs and this may have contributed to instability following PCS. Future studies may compare the use of mesocaval shunting or use of an interposition graft to minimize the impact of portal clamping in this porcine model.

The aim of the terlipressin-dosing regimen was to maintain stable PVP reduction. As lys-vasopressin is metabolized rapidly by the pig, terlipressin infusion was required to achieve stable PVP reduction. This contrasts with the terlipressin activity in humans, where clearance is slower and hence bolus terlipressin-dosing achieves stable PVP reduction. No direct side effects of terlipressin (renal dysfunction, hyponatremia, or cardiovascular effects) were observed, but these should be explored in a survival model.

Lactate and ammonia provided the most direct markers of liver dysfunction. The increased plasma ammonia observed in the PCS group supports concerns about exacerbating encephalopathy when modulating portal inflow at the time of liver surgery. There were no quantitable differences in liver histology between groups. In survival series, peak liver dysfunction does not occur until beyond day 1 post hepatectomy [29], as was demonstrated in our survival pilot. The biochemical profile immediately after 80% hepatectomy has not been described previously.

Currently there is no established therapy to prevent/treat PLF in non-cirrhotic patients. PCS [6,7], splenic artery ligation [39,40], splenectomy [41,42], and portal banding [9] have all been used to modulate post-hepatectomy PVP and prevent PLF. The degree and duration of PVP reduction required to prevent PLF in non-cirrhotic liver post-hepatectomy is unknown [4]. While surgical approaches may achieve more pronounced/sustained PVP reduction, the additional surgical morbidity associated may not be justified. Reduction in post-resection PVP with terlipressin in non-cirrhotic patients could offer several advantages over surgical strategies because terlipressin does not require additional interventions (to close the shunt or remove the portal band) and may avoid morbidity associated with surgical PVP modulation (encephalopathy and circulatory dysfunction). These benefits must be balanced against potential adverse effects that can occur at higher terlipressin doses. A stepwise approach to post-resection PVP modulation could be employed. For example, elevated PVP could initially be treated with terlipressin then if PVP is refractory or terlipressin is tolerated poorly, a surgical technique could be considered.

While previous studies have evaluated the dose, toxicity, and pharmacodynamics of terlipressin in cirrhotic patients [14–20], equivalent data in non-cirrhotic patients are limited. It is not possible to translate data from cirrhotic to non-cirrhotic patients directly because there are major differences in hepatic and systemic hemodynamics [43]. A phase I study is required to confirm the safe dose and initial proof-of-concept in non-cirrhotic patients post-hepatectomy.

There are limitations to the present study. The impact of terlipressin and PCS were evaluated in a terminal anesthetic study. It was, therefore, not possible to determine the effects of terlipressin or PCS on PLF or survival. The late hemodynamic instability that developed in the PCS group limits the ability to compare PCS with terlipressin. The mechanism for this instability is uncertain but may reflect the impact of partial portal clamping duration shunt formation.

The anesthetic agents are likely to have caused fluctuations in liver hemodynamics. However, the anesthetic protocol was developed to minimize hemodynamic changes within the liver and was standardized between groups. Future studies examining the impact of terlipressin/PCS on PLF should be undertaken in a survival series. The present paper has not presented detailed characterization of the pathogenesis of liver injury. Subsequent studies should examine differences in immunohistochemistry and gene expression between groups.

In conclusion, the PVP and PVF reduction induced by terlipressin and PCS post-hepatectomy, suggests these interventions may have a role in in the prevention/treatment of PLF. Further evaluation should be undertaken in the setting of a survival series and multicenter controlled trial.

## Clinical perspectives

- PVP can increase greatly after major liver resection, thereby increasing the risk of developing PLF, which may be prevented by maintaining PVP in the normal range.
- In this large animal study we demonstrate, for the first time, that terlipressin can prevent the increase in PVP after major liver resection in a large animal model. The effect of terlipressin was similar to that of portacaval shunting.
- The role of terlipressin in preventing PLF in humans merits investigation.

## Abbreviations

AST: aspartate aminotransferase
CVP: central venous pressure
HAF: hepatic artery flow
INR: international normalized ratio
MAP: mean arterial pressure
PCS: portacaval shunt
PLF: post-resection liver failure
PVF: portal venous flow
PVP: portal venous pressure
